# Detection of timescales in evolving complex systems

**DOI:** 10.1038/srep39713

**Published:** 2016-12-22

**Authors:** Richard K. Darst, Clara Granell, Alex Arenas, Sergio Gómez, Jari Saramäki, Santo Fortunato

**Affiliations:** 1 Department of Computer Science, Aalto University School of Science, P.O. Box 15400, FI-00076, Finland; 2 Carolina Center for Interdisciplinary Applied Mathematics, Department of Mathematics, University of North Carolina, Chapel Hill, NC 27599-3250, USA; 3 Departament d’Enginyeria Informàtica i Matemàtiques, Universitat Rovira i Virgili, 43007 Tarragona, Spain; 4 Center for Complex Networks and Systems Research, School of Informatics and Computing, Indiana University, Bloomington, USA

## Abstract

Most complex systems are intrinsically dynamic in nature. The evolution of a dynamic complex system is typically represented as a sequence of snapshots, where each snapshot describes the configuration of the system at a particular instant of time. This is often done by using constant intervals but a better approach would be to define dynamic intervals that match the evolution of the system’s configuration. To this end, we propose a method that aims at detecting evolutionary changes in the configuration of a complex system, and generates intervals accordingly. We show that evolutionary timescales can be identified by looking for peaks in the similarity between the sets of events on consecutive time intervals of data. Tests on simple toy models reveal that the technique is able to detect evolutionary timescales of time-varying data both when the evolution is smooth as well as when it changes sharply. This is further corroborated by analyses of several real datasets. Our method is scalable to extremely large datasets and is computationally efficient. This allows a quick, parameter-free detection of multiple timescales in the evolution of a complex system.

We live in a dynamic world, where most things are subject to steady change. Whether we consider the interactions between people, proteins, or Internet devices, there is a complex dynamics that may progress continuously with varying rates^1^, or be punctuated by sudden bursts^2^. Therefore, for understanding such complex systems, rich empirical data with detailed time-domain information is required, together with proper statistical tools to make use of it. Fortunately, thanks to Information and Communication Technologies (ICT) and the Web, time-stamped datasets are increasingly available. However, the development of methods for extracting useful information out of massive temporal data sets is still an ongoing task^3^.

A typical approach for studying the dynamics of a large-scale evolving complex system is to divide the temporal evolution into meaningful intervals where information of single snapshots is aggregated. The “slice” corresponding to the interval between times *t*_*n*_ and *t*_*n*+1_ then comprises the subset of elements and interactions (or events) that are active between *t*_*n*_ and *t*_*n*+1_. For instance, a slice may correspond to a group of people exchanging phone calls during an interval of one hour, or to a set of tweets containing specific hashtags posted within some time span. Once the intervals are defined, the analysis of the system turns into an investigation of the slices.

The main problem is then how to properly “slice” the data, so that the resulting slices provide the understanding we are after. Among several alternatives, one can decide to use a constant slice size corresponding to some characteristic temporal scale of the dynamics (if there is any), or a variable slice size that follows the rate of the dynamic activity. However, if we focus our attention on the evolutionary aspects of the system, it might be convenient to define the slices according to the variability of the system through time. As an example, if we want to use data on email exchanges within the company to understand the evolution of the Enron scandal, it is better to use slices that capture changes in the composition of communication groups and thus track the evolution of communication patterns, as compared to slices based solely on the rate of activity in the email communication network. Consequently, the choice of slice size will be determined both by the availability and granularity of data, and by the timescales that reflect evolutionary changes in the configuration of events.

It follows that there is a clear need for a principled way of automatically identifying suitable evolutionary timescales in data-driven investigations of evolving complex systems. This is the motivation behind the method proposed in the present paper. A proper time-slicing method should produce meaningful evolutionary timescales (intervals) describing changes in the event landscape, both when these changes are smooth and when they are abrupt. For abrupt changes, the problem becomes related to *anomaly detection*^4^,^5^ that has applications in e.g. fraud or malware detection, and in particular to *change*-*point detection* in temporal networks that comprise time-stamped contact events between nodes. For temporal networks, several ways for detecting change points have been proposed, based on techniques of statistical quality control^6^,^7^, generative network models^8^,^9^,^10^, bootstrapping^11^, and snapshot clustering^12^,^13^,^14^. Note that our approach is not specifically designed for change-point detection, but it does reveal change points as a side product of the timescale detection.

In this work, we introduce an automatic time-slicing method for detecting timescales in the evolution of complex systems that consist of recurring events, such as recurring phone calls between friends, emails between colleagues and coworkers, mobility patterns of a set of individuals, or Twitter activity about a certain topic. The method can be easily extended to systems in which interactions are permanent after their appearance (e.g. citation networks). Our approach is sequential, and consists of determining the size of each interval from the size of the previous interval, trying to maximize the similarity between the sets of events within consecutive intervals (slices). In general, there is a unique maximum: too short intervals contain few, random events whereas too long intervals are no longer representative of the system’s state at any point. Consider, e.g., a set of phone calls in a social system aggregated for a month – compared to this slice, one-minute slices would contain apparently random calls, and a 10-year slice would contain system configurations that have nothing to do with the initial slice. The method is parameter-free and validated on toy models and real datasets.

## Results

### Method for detecting evolutionary timescales

Let us consider a series of time-stamped events recorded during some period of time. Our goal is to create time slices, i.e. sets of consecutive time intervals, that capture the significant evolutionary timescales of the dynamics of the system. There are some minimal conditions that data must fulfil for this to be a useful procedure: events must be recurrent in time and the total period of recording must be long enough to capture the evolution of the system.

In this framework, the appropriate timescales detected must have the following properties: i) Evolution is understood as changes in the event landscape, i.e., intervals will only depend on the identities of events within them, not on the rate of events in time. ii) Timescales should become shorter when the system is evolving faster, and longer when it is evolving more slowly. iii) The particular times when all events suddenly change (critical times) should have an interval placed at exactly that point. This is a limiting case of (ii).

To obtain timescales satisfying the above properties, our method iteratively constructs the time intervals [*t*_*n*_, *t*_*n*+1_) according to a similarity measure between each pair of consecutive slices *S*_*n*−1_ and *S*_*n*_, where *S*_*n*_ is the set of events between time *t*_*n*_ and *t*_*n*+1_. By looking at the similarity measure as a function of time, one can detect multiple time scales. The algorithm is designed to detect the longest possible timescales in a parameter-free fashion. For discussion of timescale detection and why this is a good choice for real data, see Supplementary Information (SI), Section S1.

For the sake of clarity, assume that our method is in progress and we have a previous slice *S*_*n*−1_. We want to find the next slice *S*_*n*_ that is defined by the interval [*t*_*n*_, *t*_*n*_ + Δ*t*_*n*_). The increment Δ*t*_*n*_ is the objective variable, and is determined by locally optimizing the Jaccard index between *S*_*n*−1_ and *S*_*n*_:


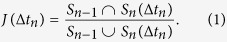


**Figure 1:**
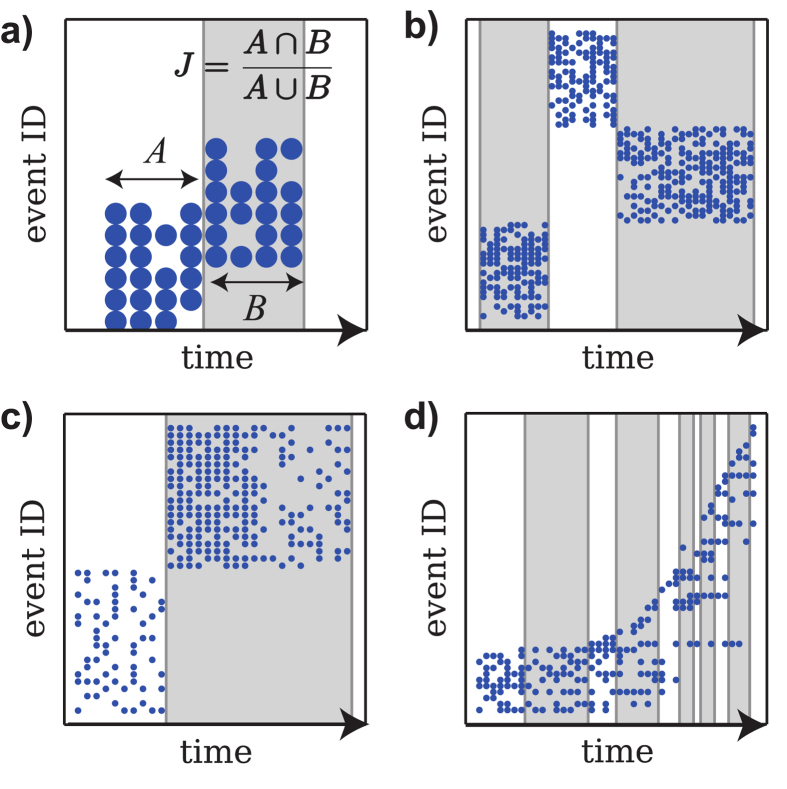
Schematic representation of the application of our method to synthetic data. In each plot, the horizontal axis represents time in arbitrary units, and the vertical axis the event ID. A dot is present at each point where a given event occurs at a given time. The gray and white segments show the detected intervals. (**a**) The Jaccard score *J* is computed for the sets of events between adjacent intervals. Intervals are adjusted to maximize *J*. (**b**) Interval identification in a trivial case. Each region contains a characteristic set of events, all distinct from those of the other regions. (**c**) The total event rate (see second interval) does not affect interval size, only the characteristic set of events does. (**d**) Example of varying interval sizes. In the first half of time, events change more slowly than in the second half, and thus intervals are longer. Because this is data with clear transitions, (**a**–**c**) have first intervals merged.

This is shown in Fig. 1(a). The Jaccard Index^15^ *J*(Δ*t*_*n*_) estimates the similarity of the two sets as the fraction of their common items (events) with respect to the total number of different items in them. In our case, the optimization process is performed by increasing Δ*t*_*n*_ until a peak for *J*(Δ*t*_*n*_) is found (see Materials and Methods). The Jaccard similarity can then be used to understand the underlying event dynamics. We stress that in the case where the same event can occur multiple times, one could also consider using weighted similarity measures, that account for the number of occurrences of the events. We discuss weighted similarity measures in the SI. In the examples we discuss results are well aligned with expectations, even though multiple event occurrences are disregarded.

This iterative process requires an initial slice, which is computed as follows: Beginning with the initial time *t*_0_, we construct two intervals [*t*_0_, *t*_0_ + Δ*t*) and [*t*_0_ + Δ*t, t*_0_ + 2Δ*t*). Repeating the process above, we find the maximum of *J*(Δ*t*) and use that to set the first two intervals at once. This initialization preserves the meaning of our slices and does not include any additional hypothesis. After computing both slices, the second one is discarded so that all subsequent intervals have the same semantic meaning, and we start the iterative process described above. Note that the method is parameter free. The Jaccard Index is used in a similar way in refs 13, 14 to assess the similarity of consecutive snapshots that are eventually grouped by means of hierarchical clustering to build relevant intervals, and in ref. 16 to study the effects of time window lengths in social network analysis.

The logic behind the method is easy to understand. Let us first consider a smoothly evolving system. We take the previous interval *S*_*n*−1_ as fixed, and try to find the next interval *S*_*n*_(Δ*t*). On one hand, if Δ*t* is small, increasing Δ*t* will very likely add events already present in *S*_*n*−1_. Thus, the numerator (intersection term) of Eq. (1) increases more than the denominator (union term), and *J*(Δ*t*) becomes larger. On the other hand, if Δ*t* is very large, the increase in Δ*t* will tend to add new events not seen in the previous interval. Therefore the denominator (union term) of Eq. (1) grows faster than the numerator (intersection term), and consequently *J*(Δ*t*) decreases. These principles combined imply the existence of an intermediate value of Δ*t* representing a balance of these factors, which is a solution satisfying our basic requirements of sliced data. If the smoothness in the evolution of the temporal data is altered by an abrupt change (critical time), the previous reasoning still holds, and a slice boundary will be placed directly at the snapshot where the anomalous behavior begins.

Our method has several technical advantages. First and foremost, it is fast and scalable. The method is *O*(*N*) in the total data size *N* (e.g. number of distinct events), if intervals are small. Efficient hash table set implementations also allow linear scaling in interval size, and therefore extremely large data sizes can be processed. The data processing is done online: only the events from the previous interval must be saved to compute the next interval. There are no input parameters or *a priori* assumptions that must be made before the method can begin. The input is simple, consisting simply of (event_id, time) tuples. The method finds an initial intrinsic scale to the data, where each interval represents roughly the same amount of change.

### Validation on synthetic data

First we have generated synthetic temporal data examples to be analyzed with our method. The data displays several common configurations of the temporal evolution of a toy system. Figure 1 shows some examples and the timescales that our method detects. In Fig. 1(a) we sketch the details of the method. Figure 1(b) shows three clearly distinguishable sets of events, which are correctly sliced into their respective timescales. Within each interval, events repeat very often, but between intervals there are no repetitions. Figure 1(c) shows that the rate of the short-term repetitions does not matter from our evolutionary point of view. The second interval has the same characteristic events at all times, so no evolutionary changes are observed. Figure 1(d) shows a continually evolving system. For the first half, the long-term rate of evolutionary change is slow (events are repeated for a longer time before dying out), and thus the intervals are longer. Thereafter, the process occurs much faster, and thus the intervals are shorter.

We have also validated our method on more complex synthetic data designed to reveal non-trivial but controlled evolutionary timescales. We choose a dynamics where there is a certain fixed number of events which, at every time, might be active or inactive (short-term repetition). We impose that the volume of active events per time unit must be, on average, constant. Additionally, to account for the long-term evolution, we change the identities of the events at a (periodically) varying rate. Low rates of change, that is, changing the identities of only a few events at any time, should result in large time intervals for the slices, while faster rates lead to high variations in the identities of the events and should produce shorter slices.

**Figure 2:**
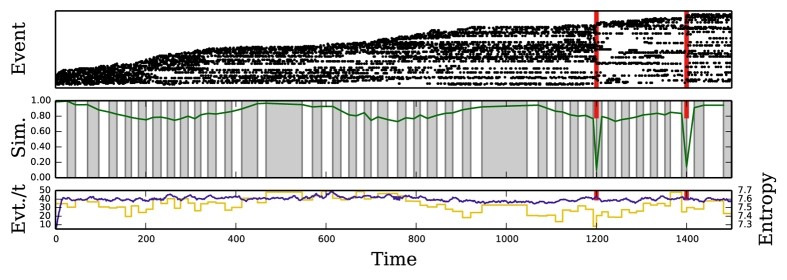
Artificial data with changing event turnover rate with period *τ* = 500. (Top) Representation of events. Events have been ordered to show the envelope of the dynamics. Events change at the slowest rate at *t* = 500, 1000, and there are critical points at *t* = 1200, 1400. For clarity, only 10% of events are shown. (Middle) Detected intervals (gray/white regions), and inter-interval similarity (plotted green lines) for the slicing algorithm. We can see that the internal length reacts to the rate of change of events, and that the critical points correspond to intervals boundaries and to large drops in similarity. The similarity also reflects the rate of change of events at other times. (Bottom) Event rate (dark blue) and interval entropy (light yellow) over time. We see that overall event rate is constant and cannot be used to detect dynamical properties. We also see that each interval contains about the same amount of information, 7.3 to 7.7 bits.

We check our method on this benchmark using a periodic evolutionary rate, with period *τ* = 500 (see Materials and Methods). In Fig. 2, the method shows the desired behavior, namely, it produces short intervals in regions where change is fastest (*t* ≈ 250, 750) and longer intervals for low variability of events, i.e. slow evolution (*t* ≈ 0, 500, 1000). Most notably, the Jaccard similarity is seen to reflect these dynamics, too. When change is slowest, the inter-interval similarity is high, and vice versa. These similarities could be used to perform some form of agglomerative clustering to produce super-intervals representing longer-term dynamics. We also show the Shannon entropy within each interval. We see that our method produces intervals containing roughly the same amount of information.

At times *t* = 1200 and *t* = 1400, we add two “critical points”, when the entire set of active events changes instantly. This is the limiting case of evolutionary change. As one would expect, slice boundaries are placed at exactly these points. Furthermore, the drastic drop in similarity indicates that unique change has happened here, and that dynamics across this boundary are uncorrelated.

### Analysis of real temporal data

In this section we apply our approach to real-world data, where any true signal is obfuscated by noise and we do not have any information about the underlying dynamics.

**Figure 3:**
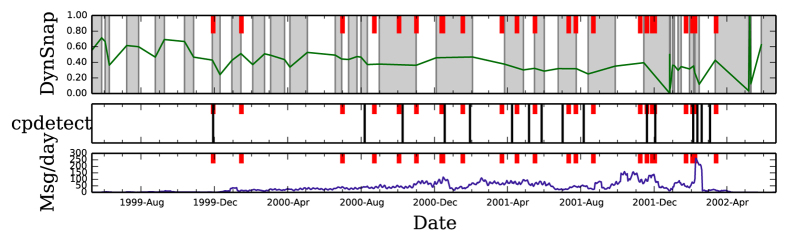
Comparison of our method and the change point detection method of ref. 8 on the Enron email data. Thick red lines indicate events from the company’s timeline. (Top) Results of our method on the network, in the same format as Fig. 2. (Middle) Results of the *cpdetect* method on the network. Dark vertical lines mark change points. (Bottom) Event density of the network. We see that both methods align with the company’s events to some degree. Our method provides more fine-grained intervals, not just the major points, and is capable of more accurately aligning with the real events since it does not have predefined window sizes as an input.

We start with the Enron email dataset (see Materials and Methods). This dataset was made public during the legal investigation concerning the Enron Corporation, which ended in the bankruptcy and collapse of this corporation^17^. Here, each event is an email communication sent or received by any of the senior managers who were subsequently investigated. During the course of the investigation, there were major structural changes in the company (see SI). Figure 3 shows the results of applying the method to the Enron data. Generally, the intervals are of the order of several weeks, which appears to be a reasonable time frame for changes in email communication patterns. As expected, we see that the detected intervals do not simply follow changes in the rate of events, but rather in event composition. When comparing interval boundaries to some of major events of the scandal (exogenous information, shown as red vertical lines), we see that the interval boundaries often align with the events; note that there is no a priori reason for external events always to result in abrupt changes in communication patterns. Additionally, we present the results of the application of the change point detection method of Peel and Clauset (*cpdetect*)^8^. Unlike *cpdetect*, our method reveals the underlying basic evolutionary timescale (weeks), rather than only major change points. On the other hand, *cpdetect* requires a basic scale as an input before its application (in this case, one week). Performing a randomization hypothesis test, we find evidence (at significance *p* = 0.0194) that *cpdetect* change points correspond to lower-than-random similarity values within our method (see SI).

**Figure 4:**
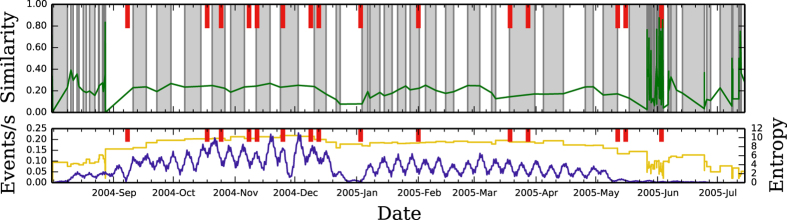
Slicing of the MIT reality mining dataset. In this dataset, an event is considered the (from, to) pair of Bluetooth scanning, representing two devices being located within proximity of each other. The times of major events are known, such as important conferences or start/ends of semesters (see SI). We see that the slicing can find such major events, including the beginning of the 2005 semester and the beginning of classes at 2005-Feb.

Next, we focus on the MIT Reality Mining personal mobility dataset. In this experiment, about 100 subjects, most of them affiliated to MIT Media Lab, have cellphones which periodically scan for nearby devices using the Bluetooth personal area network^18^. Here, events correspond to pairs of devices being in Bluetooth range (i.e. physically close). Note that we do not limit the analysis to interactions only between the 100 subjects, but process all ≈5 × 10^4^ unique Bluetooth pairs seen (including detected devices not part of the experiment), a total of 1.8 × 10^6^ distinct data points. The major events (see SI) correspond to particular days in the MIT academic calendar or internal Media Lab events. In Fig. 4 we plot the time slices generated by our method. Again, we see that the basic evolutionary timescale is about one week. We see a correlation with some of the real events, such as a long period during winter holidays (late December) and Spring Break (late March). At the beginning (August) and end (June) there is a minimal data volume, assimilable to noise, which precludes the method to find reliable timescales.

In our last example, we look at the social media response to the semifinals of the UEFA Champions League 2014–2015, a major European soccer tournament. We collected data from Twitter looking for tweets containing the hashtag #UCL, the most widely used hashtag to refer to the competition. As all captured tweets contain the hashtag #UCL, we define events by considering the other hashtags that a tweet may contain.

**Figure 5:**
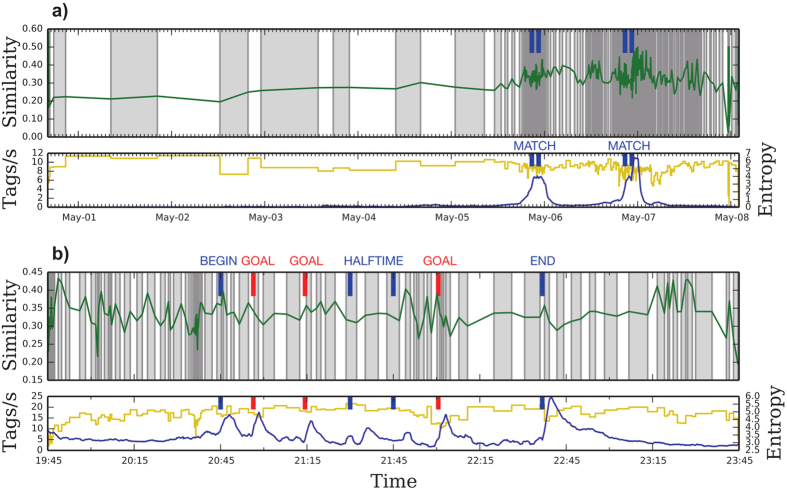
(**a**) Slicing of Twitter hashtag sequences generated during UEFA Champions League 2014–2015 football tournament. The format of the figure is as in Fig. 2. In this data all tweets corresponding to the #UCL hashtag are captured, so the rest of the co-occurring hashtags are taken as our events. The two increments in the event rate represent the two matches, which are captured through very fine intervals in the data slicing. (**b**) First semifinal of UEFA Champions League 2014–2015, between Juventus and Real Madrid. The format of the figure is as in Fig. 2. The game runs from 20:45 until 22:30, with the halftime from 21:30 until 21:45. We see sped-up activity around the goals in the slicing (top) that is not simply explained by the event rate (bottom). When goals are scored (20:53, 21:12 and 21:55) there is a much faster topics turnover.

If a tweet contains more than one other hashtag, each of them is considered as a separate event. For example, if at time *t* there is a tweet containing #UCL, #Goal and #Football, we consider that we have two events at time *t*, one for #Goal and the other for #Football. In Fig. 5(a), we see a one-week span around the two first semifinal games. Because Twitter discourse is rather unstructured, intervals are very small, comprising at most a few hours. During the games themselves (May 5, 6 evenings), we see extremely short intervals representing the fast evolution of topics. In Fig. 5(b), we zoom in the first semifinal game. As the game begins, intervals become much shorter as we capture events that did not manifest in the previous games to the match. We see other useful signatures, like the rapid dynamics at the beginning of the second half (21:45) and at the time of the game-winning goal (21:58).

One could wonder if our method detects something in our data, beyond what one would expect in random data. To study this, we do a shuffling test. For each dataset, we compare the initial data with 100 randomized cases. Randomization is performed by shuffling all event IDs while preserving the same set of timestamps. For the base case, we calculate the widths of all intervals. We use a Mann-Whitney U test to compare this distribution to the distribution of all widths found in 100 resamples. The resulting p-values are listed in Table 1 and show that our results are clearly distinguishable from those found in random event streams datasets.

## Discussion

Complex systems are inherently dynamic on varying timescales. Given the amount of available data on such systems, especially on human behavior and dynamics, there is a need for fast and scalable methods for gaining insight on their evolution. Here, we have presented a method for automatically slicing the time evolution of a complex system to intervals (evolutionary timescales) describing changes in the event landscape, so that the evolution of the system may be studied with help of the resulting sequence of slices.

Our method provides the starting point of a pipeline analysis. Many time-varying data analysis techniques require segmented data as an input, and if the intervals are decided according to some fixed divisions, the boundaries will not be in the optimal places. Our method places snapshot boundaries in principled locations, which will tend to be the places with the highest rate of change - or actual sharp boundaries, if they exist. After this process, the sliced data can be input to other methods which further aggregate them into even higher orders of structure while respecting the underlying dynamics.

Our code for this method is available at ref. 19 and released under the GNU General Public License.

## Methods

### Identification of optimum interval

The value of Δ*t* is searched from the raw data, and various strategies can be used. A simple linear search suffices, but is inefficient at long timescales. An event-driven approach checks only Δ*t* intervals corresponding to actual events, but this too is inefficient at long timescales. An ideal approach, and the one adopted in this paper, is to use exponentially spaced intervals (see SI). The shortest Δ*t* searched is taken from the next event in the data.

### Temporal benchmark

The proposed benchmark has a periodic activity change in time, while having a uniform event rate. A universe of *N* = 1000 event IDs is created. Upon initialization, each event is independently placed into an activated status with probability *q*. At each time step, each active ID creates an event with probability *p*. Also, at each time step (and before event creation), a fraction *c*(*t*) of event IDs are picked. Each of these IDs has its active status updated, being made active with probability *q* regardless of its previous state. This preserves the mean total number of activated IDs as *qN* at all times. At each time step, on average *pqN* events are expressed. Thus, by looking at simply the rate of events, the model is completely uniform and there is a constant average event rate in time.

The changeover rate *c*(*t*) is periodic, however, obeying


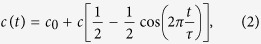


with *c*_0_ being the minimum changeover rate, *c* the scale of changeover, and *τ* the period. Thus, at some points, the IDs of the expressed events is changing more rapidly than at other times, and this is the cause of longer and shorter intervals.

When there is a critical event at time *t*_crit_, we exceptionally set *c*(*t*_crit_) = 1 at that time. This produces a state of the system uncorrelated from the previous time. According to the model, this changeover occurs before the time step, which matches with placing a new interval at *t*_crit_ in our half-open segment convention. However, the total universe of event IDs stays the same, and there is no statically observable change in behavior.

In this work, we use *N* = 1000, *τ* = 500, *p* = 0.2, *q* = 0.2, *c*_0_ = 0, and *c* = 0.01.

### Shannon Entropy

The Shannon entropy ref. 20 is calculated by 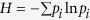 over a series of events *i* with corresponding probability *p*_*i*_.

### Enron temporal data

The Enron Email Dataset consists of emails of approximately 150 senior managers of the Enron Corporation, which collapsed in 2001 after market manipulation was uncovered, leading to an accounting scandal^17^. We have all bidirectional email communications to and from each key person with a resolution of one day. An event is the unordered pair (source, destination) of each email. Our particular input data is already aggregated by day, therefore each event is only repeated once each day, with a weight of the number of mails that day. SI Tables S1 and S2 lists the basic properties and SI Table S3 lists the major events of this dataset.

### Reality mining

The Reality Mining dataset covers a group of persons affiliated with the MIT Media Lab who were given phones which tracked other devices in close proximity via the Bluetooth personal area network protocol^18^. An event is defined as every ordered pair (personal_device, other_device). We only have data from the personal devices of the 91 subjects who completed the experiment, not full ego networks. The data contains 1881152 unique readings from 2004 August 8 until 2005 July 14, with most centered in the middle of the academic year. SI Table S4 lists basic properties and SI Table S5 lists the major events of this dataset^21^.

### UCL hashtags

In this dataset, we scrape Twitter for all hashtags containing #UCL, referring to the Europe Champions League tournament. We scrape between the dates of 30 April 2015 and 08 May 2015, covering the two first semifinal games of the tournament. An event is any hashtag co-occurring with #UCL, except #UCL itself. If there are multiple co-occurring hashtags, each counts as one event. All hashtags are interpreted as UTF-8 Unicode and case-normalized (converted to lowercase) before turning into events. SI Table S6 lists basic properties and SI Table S7 lists the major events.

## Additional Information

**How to cite this article**: Darst, R. K. *et al*. Detection of timescales in evolving complex systems. *Sci. Rep.* **6**, 39713; doi: 10.1038/srep39713 (2016).

**Publisher's note:** Springer Nature remains neutral with regard to jurisdictional claims in published maps and institutional affiliations.

## Supplementary Material

Supplementary Information

## Figures and Tables

**Table 1:** Test of results on real datasets with respect to a baseline consisting of a random reshuffling of the events.

	*p*-value	width, mean ± std baseline	width, mean ± std reshuffled	*N*_*o*_	*N*_*r*_
MIT reality mining	2.75 × 10^−16^	1.5 × 10^5^ ± 2.5 × 10^5^ s	3.3 × 10^5^ ± 6.2 × 10^5^ s	191	9088
Enron	3.0 × 10^−27^	17 ± 12 days	153 ± 182 days	69	783
Twitter	2.2 × 10^−113^	1248 ± 5410 s	3775 ± 6086 s	513	16961

The p-values refer to the likelihoods that the width distribution of intervals of our method on the original data is the same as the width distribution of randomized data (100 resamplings). *N*_*o*_ and *N*_*r*_ refer to the total number of detected intervals on the original data and in all 100 resampled trials respectively. The width distributions are extremely broad, thus standard deviations are large relative to the means.
