# Supplementary material for: Detection of timescales in evolving complex systems
Source: Sci Rep. 2016 Dec 22;6:39713. doi: 10.1038/srep39713 (PMC5177884; doi:10.1038/srep39713)
Supplement: Supplementary Information [file srep39713-s1.pdf]

Detection of timescales in evolving complex systems
Supplementary Information

Richard K. Darst,[1] Clara Granell,[2] Alex Arenas,[3] Sergio Gómez,[3] Jari Saramäki,[1] and Santo Fortunato[1,4]
[1]*Department of Computer Science, Aalto University School of Science, P.O. Box 15400, FI-00076, Finland*
[2]*Carolina Center for Interdisciplinary Applied Mathematics, Department of Mathematics, University of North Carolina, Chapel Hill, NC 27599-3250, USA*
[3]*Departament d'Enginyeria Informàtica i Matemàtiques, Universitat Rovira i Virgili, 43007 Tarragona, Spain*
[4]*Center for Complex Networks and Systems Research, School of Informatics and Computing, Indiana University, Bloomington, USA*
(Dated:)

S1. ALGORITHM DESCRIPTION

The algorithm for detecting the evolving timescales of time-varying data is implemented in different layers. Each layer is reasonably independent of others, allowing them to be improved or replaced independently of each other. This allows the method to be easily adapted and improved for new types of data. This document describes each of the possible variations of each layer of implementation. Please note that this document is designed to explain the algorithm components from a scientific viewpoint. It is not a usage manual of the code, which is found on the project site.

The algorithm is designed in the following layers:

1. Data input (See user manual)
2. Data representation (Sec. S1 B 1)
3. Similarity measures (Sec. S1 C)
4. The core segmentation process (Sec. S1 D)
5. Choosing Δt to test at one iteration (Sec. S1 E)
6. Maximum finding (Sec. S1 F)

Code for the method is available at `https://github.com/rkdarst/dynsnap`.

A. Data and timescales

Before the method is applied to data, it is important to understand the necessarily qualities of input data and how timescales are detected. The method is by default suited to most real data in a parameter-free fashion, but there can be non-intuitive behavior for data with long-term self similarity, such as completely periodic artificial data.

The purpose of this method is to segment events into time-intervals so that the spacing of intervals corresponds to the similarity of events within those intervals. This is captured by looking at the similarity of events within intervals. An interval is too short if there is not enough time to get a characteristic set of events within it. An interval is too long if, by some definition, the start and end of the interval are different in terms of characteristic events. All else being equal, we would rather take longer intervals. Data must have two properties in order to be sliced. First, events must repeat on a short time scale. That is, events repeat in time, so that the same event can be captured in two adjacent intervals, or else intervals can not be similar. This is our smallest possible slicing time. If events do not recur, then our method can not be used. On the other hand, there must be a long time scale at which events do *not* recur. If events did always recur at every time scale, then the longest time scale of self-similarity covers the entire time. In this case, any equal-size slicing of the interval would be acceptable. There are two ways this long-term change can be understood. One is if some events become active, turn on and off for some time, and then eventually deactivate. That is, you can identify some time range when an event occurs, and then forever before and after that time, that event is not seen. Alternatively, there can be a finite universe of events, and the events are extremely bursty, so that there are very long inactive periods between phases of activity.

Our method can detect different timescales. If there are different timescales, then $J(\Delta t)$ (Sec. S1 F) for each slice will reveal them. There is then the question of selecting which of these timescales the algorithm should return for any

given run of the code. The algorithm described here and the code released along with this article adopts the strategy that, by default, the longest possible timescale should be detected. There are options to find shorter timescales, and a detailed discussion exists within the code manual at `doc/Manual.rst`, and in Sec. S1 F and Sec. S1 F 4.

To use this method on data which does not meet the criteria above, in particular the "long term change", timescale options will be needed. In particular, consider the case of periodically repeating data, especially artificial data that has no random noise. There is a short term similarity timescale, smaller than the period of repetition. This is the most useful timescale to detect. Detecting this timescale would allow one to detect the components within the repeating pattern. However, there is also a similarity at a long term timescale. Detecting this timescale would override and hide all internal similarity. Since this similarity extends to all time and our method by default tries to find the longest possible timescale, we would find the (less interesting) result that all time is similar. Another way to see this is that there is as much, if not more, similarity between the first 1/3rd and last 1/3rd of data as there is within each period. (Note that this can be used as a test for data where time scale adjustment is needed). Thus, our method correctly detects the longest timescale as covering all time. However, note that any small amount of non-repeating random noise will destroy this long-term similarity, and the method then detects some shorter time scale. This is why we say that our method works well and without parameters on real data - random noise destroys long-term similarity, so detecting the longest time scale returns the expected result.

B. Preliminaries

All time ranges are considered half-open intervals $[t_1, t_2)$. Within an interval, events are aggregated to produce "sets of events" which characterize the interval. The core goal of the slicing algorithm is to produce adjacent intervals that have different sets of events, but that are not too dissimilar.

1. Data and interval representation

At the lowest level, all data is a multiset of `(time, id, weight)` tuples for each event. The variable `time` refers to the time at which an event has happened, `id` stands for the unique ID of the event, and `weight` stands for the number of occurrences of that particular event at that particular time. This conforms a multiset because duplicate events at the same time and ID are allowed. If data is unweighted, all weights can be considered to be (and stored as) 1.

When using data in an unweighted fashion, then the set of events is a regular (non-weighted, non-multiset) set containing the IDs of every event present within the interval.

When using data in a weighted fashion, a set of events is a weighted set containing event IDs for every event present within the interval, where each event has an associated (non-negative) weight. If the original data has only unit weights (or was originally unweighted), then the event weights reduce to the counts of events within the intervals. The weights are the sum of weights of all events of that ID within that interval.

Note that a weighted set can be converted to unweighted by dropping all weights. Conversely, if unweighted data is made weighted, the weights count the number of events present.

C. Similarity measures

The various similarity measures are defined as a function between two (possibly weighted) sets of events as defined above, with a resulting value in the range $[0, 1]$. A 1 similarity defines a perfect match while 0 indicates no similarity.

In the following examples, consider two intervals A and B. Here we are loose with terminology and use the terms A and B interchangeably to refer to the interval itself as well as to the set of events within the intervals. In the remainder of this document, we use J to refer to a generic similarity measure, even if the symbol itself refers to the Jaccard score.

1. *Unweighted Jaccard*

This measure calculates the similarity between unweighted sets of events. It makes use of the standard Jaccard score,

$$J(A,B) = \frac{|A \cap B|}{|A \cup B|}. \tag{S1}$$

In this formulation, the Jaccard score is 1 if two intervals have the same elements regardless of the number of occurrences of those elements within the intervals.

2. *Weighted Jaccard*

The following is the extension of the Jaccard score to the intersection and union of weighted sets. Weighted sets are defined by real-valued indicator functions w_i representing the weight of each element within the set. Element i has a weight of w_i. Any element of weight zero is considered to not be contained in the set and can be removed. Conversely, any element not present in the set has a weight of zero. A weighted union is defined to have elements of

$$w_{U,i} = \max(w_{A,i}, w_{B,i}) \tag{S2}$$

over all elements in either A or B. Here, $w_{U,i}$ is the indicator function for the union, and respectively $w_{A,i}$ and $w_{B,i}$ for the sets A and B. A weighted intersection is defined to have elements of

$$w_{I,i} = \min(w_{A,i}, w_{B,i}) \tag{S3}$$

with components analogous to Eq. (S2). With these definitions for the intersection and union, the weighted Jaccard score is computed as in Eq. (S1).

It is worth noting that the weighted Jaccard score introduces a bias towards equal-size sets with equal element counts. Thus, there is some “inertia” in interval sizes and can not adapt to changing timescale quickly.

3. *Cosine similarity*

The weighted sets can be considered sparse vectors, allowing us to use the cosine similarity. Defined in terms of sets, the cosine similarity is

$$C(A,B) = \frac{A \cdot B}{|A||B|} = \frac{\sum(w_{A,i} w_{B,i})}{\sum(w_{A,i}) \sum(w_{B,i})}. \tag{S4}$$

The cosine similarity of 1 indicates perfect match between events and relative event counts, but does not require the same number of total events. Thus, the cosine similarity takes into account event counts in a more flexible way than the weighted Jaccard score. The cosine similarity can be 1 if the sets are of unequal sizes, as long as the relative distribution of event weights is the same.

4. *Unweighted cosine similarity*

The unweighted cosine similarity is defined as

$$C(A,B) = \frac{|A \cap B|}{\sqrt{|A||B|}} \tag{S5}$$

This is the analog of Eq. (S4) when applied to unweighted sets. It has many of the same advantages and disadvantages as the unweighted Jaccard score.

D. The slicing process

The slicing of the data is the core of the algorithm. It provides an efficient, one pass, linear time method of segmenting the groups of events in intervals where each interval i comprises the time range within the half-open interval $[t_i, t_{i+1})$. The interval size is $\Delta t_i^* = t_{i+1} - t_i$. Our general procedure is:

1. Begin with some initial time t_0. This is either the time of the very first event, or some specified time if one wishes to segment only a portion of the time period.
2. Find the optimal Δt_0^* for the first interval. Various values of Δt are tried (see Sec. S1 E), and the optimum is the value which maximizes the similarity $J(\Delta t)$ (see Sec. S1 F). The interval is then set to $[t_0, t_0 + \Delta t_0^*)$.
3. Repeat the previous steps until all data is treated, or until we reach a specified stop time. We go through time by setting the start of the next interval at the end of the previous, $t_i = t_{i-1} + \Delta t_{i-1}^*$.

1. *Initial step: calculating the size of the first interval*

We begin with an initial time t_0, which is the lower bound of the first interval. If this is not provided by the user, it is the time of the first event. A test sequence of Δts is generated via one of the methods described in Sec S1 E. For each Δt generated, we compute the intervals $A(\Delta t) = [t_0, t_0 + \Delta t)$ and $A'(\Delta t) = [t_0 + \Delta t, t_0 + 2\Delta t)$. These will be the next two intervals of width Δt after t_0. We then compute our similarity score J (Sec. S1 C) between A and A' as a function of Δt:

$$\begin{aligned} J(\Delta t) &= J\left(A(\Delta t), A'(\Delta t)\right) \\ &= J\left([t_0, t_0 + \Delta t), [t_0 + \Delta t, t_0 + 2\Delta t)\right). \end{aligned} \tag{S6}$$

Eq. (S6) is maximized as a function of Δt to produce Δt^*, our optimal interval size (see Sec. S1 F for a detailed explanation of this procedure). Once Δt^* is found, the first interval A is set to $[t_0, t_0 + \Delta t^*)$. This interval is now fixed, the starting time is updated to $t_1 = t_0 + \Delta t_0$ and we proceed to the propagation step.

Merging of the first two intervals. This method provides an option which consists in merging the initial intervals. In this process, in the initial step, the first two intervals detected (A and A') are merged into one double-sized interval. Continuing from Sec. S1 D 1, after Δt^* is calculated, the first interval A is set to $[t_0, t_0 + 2\Delta t^*)$, and the new starting time is set to $t_0 = t_0 + 2\Delta t$. If this option is chosen, this process is done at the beginning of the data slicing as well as after any critical events (which cause a restart in the slicing process as explained in Sec. S1 F 5). This avoids discarding the second interval $[t_0 + \Delta t^*, t_0 + 2\Delta t^*$, but causes the combined first interval to have a different size distribution from subsequent calculations.

In cases where the data has only sharp transitions, this merge process is advantageous, since the first two intervals will probably be more similar than what comes after it. However, in smoothly varying data, merging should not be done because it changes the meaning of the first interval relative to others. This is noticeable when the first interval is twice as long as others. In the end, this decision must be made with external information, and it should be used only if needed. Note that this decision is only relevant at the beginning of time, when the method is first learning relevant timescales.

This is done in main text Fig. 1(a–c), where the data has sharp, clear transitions. Without this process, existing boundaries stay the same, but each interval is divided into two. Thus, there are pairs of $J = 1$ intervals (self similar), followed by pairs of $J = 0$ intervals (critical events, Sec. S1 F 5).

2. *Propagation step*

Given our previous interval A and starting time t_i at the end of A, we proceed to construct the next interval in a similar fashion to the initial step. We generate our series of Δts and construct a series of intervals $B(\Delta t) = [t_i, t_i + \Delta t)$ for each Δt. Analogously to the initial step, we compute the similarity score as a function of Δt,

$$\begin{aligned} J(\Delta t) &= J\left(A, B(\Delta t)\right) \\ &= J\left(A, [t_i, t_i + \Delta t)\right). \end{aligned} \tag{S7}$$

The difference to the initial step is that the first interval is fixed, and only the second is changing. We choose the Δt^* which maximizes $J(\Delta t)$. The next interval is then fixed as $B = [t_i, t_i + \Delta t^*)$.

We repeat the propagation step indefinitely, until the intervals reach the end of the data and all events are included. For each iteration, we take the A as the previous interval, and begin at the next start time $t_i = t_{i-1} + \Delta t_{i-1}^*$.

E. Δt generation

There are various methods to choose the Δts to test in the optimization process of the previous section. We must explicitly generate some values, because this is a numerical optimization. It is important to do this cleverly, or else the method can become very inefficient. We would rather not test every possible Δt value, or test values too far in the future. We would prefer to check small Δts that are close together, but they should be spaced further apart at long Δt. We would rather check small Δt values first, since smaller intervals have fewer events to test, and thus are faster to compute.

Also, there is a major opportunity for optimization. As long as Δt increases, interval sets can be generated by simply adding (via set union) new events (between Δt_{n-1} and Δt_n) to the previous interval set. This is a very efficient operation, and makes each individual iteration $\mathrm{O}(\Delta t_{\mathrm{max}})$, assuming that Δt only increases.

Since we do not have an *a-priori* knowledge of the minimum or maximum reasonable interval size, these are structured as generators of Δt values, returning an infinite sequence. At a certain point, the algorithm detects that we have searched enough, and that it is likely that we already obtained the peak in similarity we were looking for, which causes the generation of Δt to stop (as described in Sec. S1 E 5).

The methods described below are currently defined in the code. Better methods could be implemented in the future, including an actual bisection to find the optimum. Only the logarithmic method is fully developed for actual use.

1. Linear scan mode

In this mode, the values $m + 1d, m + 2d, m + 3d, ...$ are iterated. The parameter d is the step size (1 by default), and m is the minimum step size (default to the same as d). This method does not automatically adapt to the data scale, thus reasonable values of step size and minimum step size must be provided. Further, this method is inefficient for data with a very long timescale, or data that evolves in very different timescales.

2. Logarithmic scan mode

In this mode, we begin with a base scale of

$$m = 10^{\lfloor \log_{10}(t_e - t_i) \rfloor} \tag{S8}$$

where $t_e - t_i$ is the time to the next event after the interval start time t_i. Thus, m is the greatest power of 10 less than the time to the next event. This allows the scan mode to adapt to the actual data sizes. Then, we return the sequence of values:

$$1m, 2m, \ldots, 99m, 100m, 110m, 120m, \ldots, 990m, 1000m, 1100m, \ldots, \tag{S9}$$

which produces a logarithmic time scale with reasonable precision at all points.

3. Event-based scan mode

In this mode, for every distinct event time $t_e > t_i$, we return the corresponding $\Delta t = t_e - t_i$. Recall that t_i is the start of the interval. In this way, every time step with presence of events is tested. This mode offers the greater precision in aligning intervals with events, but can be inefficient for very large datasets at very long times, where each individual event is unlikely to have an effect on the Jaccard score.

4. An ideal mode

An ideal method would combine parts of the above methods. It would begin with a logarithmic scanning, but ideally with some fixed multiplier such as 1.01. The next time is found by $\Delta t_{\mathrm{next}} = \lceil 1.1 \Delta t \rceil$. Here, the ceiling operator $\lceil \cdot \rceil$ means *the time of the next event equal to or after the given time.* This allows a logarithmic increase in time, while always aligning with actual events and skipping non-present events. After a maximum is found, we would backfill with a bisection algorithm to find the exact event which produces a global optimum for the similarity peak.

The downside to this method is that it requires many searches through our data to find the event-ceiling. This is implemented as a fast database search, but still requires extra operations. Also, the bisection stage would ideally

need to be able to increment sets both forward and backwards in time. Currently, the process of set construction is optimized to incrementally build up the sets while moving forward in Δt. Going backwards is possible with weighted sets (though this procedure needs to be done with caution, watching out for floating point errors), but with unweighted sets this operation is not possible. This biases us to do a more thorough scan going only forward in time. The current logarithmic implementation is seen as a good trade-off between simplicity, accuracy, generality, and computational performance.

5. *Criteria for stopping the Δt generation*

The above methods do not specify when to stop searching new Δt values (except when using greedy maximum finding, as we will see in Sec. S1 F 3). The intuition is that there will always be some maximum, independently of the number of values of Δt that we choose to scan. In order to have a good chance of finding the global optimum, we need to carefully adjust how many time steps we will search after the latest found peak. If we decide to search in a small time window, we have great chances to get trapped in a local maxima. Instead, if we opt for scanning a very large time window, we will certainly sacrifice the computational efficiency. As usual, a balance between these two opposite cases is desired. We achieve this by continuing to scan until we have tested all $\Delta t < 25\Delta t^*$ and Δt less than 25 times the previous round's Δt^*, if there is a previous interval. As mentioned, the multiplier can be adjusted lower for better performance or higher for less risk of missing future peaks, but this is the subject of further research.

F. Maximum finding

The Δt values are given by one of the methods from Sec. S1 E, and we wish to find the optimum value of Δt^* such that it maximizes $J(\Delta t)$. The first consideration is that there will likely be many local maxima. Some will be caused by general fluctuations in the data. However, when scanning on a larger scale, we may see different local maxima, which can be interesting in their own right, because they may indicate different time scales of the system. These can be seen by plotting $J(\Delta t)$ as a function of Δt, primarily for the first interval. Sec. S1 F 4 discusses some methods of adjusting the timescale detection to find other maxima.

Then the question is under what conditions do we expect non-trivial maxima to exist. When there is a long-term evolution of the system (events active at $-\infty$ are different from those at $+\infty$), then we will obtain a decrease in similarity as Δt becomes longer. In this case, there will not be a maximum. However, if there is not a long-term evolution (i.e. for each event ID, that event has the same probability of occurring at any point in time), then the similarity $J(\Delta t)$ may continually increase. In this case, the basic assumptions of our method are violated, but the answer is correct anyway: as all times are statistically self-similar, we expect one giant interval covering all time because there are no sub-divisions of distinct character.

In brief, the process of maximum finding works as follows. For each Δt, we calculate the similarity $J(\Delta t)$. We search for the maximum value of $J(\Delta t)$ in an on-line fashion. The Δt iteration (see Sec. S1 E) produces a continuous stream of Δt values. After each Δt is produced, $J(\Delta t)$ is calculated (as in Sec. S1 C). Then, the list of all Δt and $J(\Delta t)$ are examined by the methods that we will present next in this section, which return Δt^*, the optimum interval size. This Δt^* is fed back to the stop criteria in Sec. S1 E 5 and used to decide when we should cease exploring further Δt. Once the stop criteria is met, the iteration stops and the final Δt^* is known.

1. *Longest*

This method is our standard approach. We search for the maximum similarity value of $J(\Delta t)$ from all possible Δt values. If there are multiple values of Δt with the same maximum, we pick the greatest one as Δt^*.

2. *Shortest*

This is similar to “Longest”, but choosing the shortest Δt.

3. Greedy

In this method, as soon as $J(\Delta t)$ decreases for the first time, we stop testing other values. This method is much more efficient in terms of computation time. While there is usually one clear peak, there are often local fluctuations which cause this method to give a local maximum far before the global maximum. If the number of events is large enough to reduce the effects of fluctuations, or if the highest computational performance is needed for streaming data, this method could be useful. If this method is used, the stop criteria of Sec. S1 E 5 is unneeded.

4. Detecting other timescales

One of the basic assumptions of this method is that the similarity score increases, and then decreases. We would hope that as the similarity starts going down, we can notice a peak. The peak finders, as described above, do not find multiple local maxima. This is because choosing a peak requires some heuristic for how significant of a peak should be detected. The two extremes are represented by the "longest"/"shortest" peak finders (scan forward in time as much as possible, find peak as global maximum in scanned area) and "greedy" (stop at the first decrease, as soon as any maximum is found; in other words the first local maximum). This corresponds to finding the longest similar time scales and shortest similar time scales.

Peak factor. The "peak factor" is a way to balance these. It is implemented only for the "longest" and "shortest" maximum finding methods. Using this option, once $J(\Delta t_{\max})$ drops to less than peak_factor $\times$ $J(\Delta t^{*})$, then we stop searching any longer times and return Δt^{*}. This provides a way to break out of the search after the first peak.

Pastpeak factor. The factor of 25 present in Sec. S1 E 5 can be adjusted. This limits the forward search time and provides another way to limit the timescale searched.

Pastpeak max, pastpeak min, search min. One can directly set the time scales searched. One can set a maximum or minimum amount of time to search past Δt^{*} (as in Sec. S1 E 5). One can also set a minimum amount of time to search past $\Delta t = 0$. This may be useful to avoid the effects of extreme fluctuations in J at small Δt. Unlike the other options, these require a pre-existing knowledge of likely time scales within the data. Because of this, these options are not used in this work or enabled by default. The existing options work to achieve the goal of detecting the longest possible timescales without needing any parameters.

5. Critical event detection

At some times (critical times), the character of the active events in the entire data changes instantly. When this happens, comparison with the previous interval will give bad results, since similarity is by definition going to always be low. Similarity scores could also remain zero, if there is no overlap in the set of events before and after the critical time. Thus, the signature of critical events is low but continually increasing similarities with no peak found. At this point, the algorithm will tend to produce longer and longer intervals, pointlessly trying to maximize similarity and eventually return too large intervals. At this point, using the "initial step" process (see Sec. S1 D 1) is better than using the propagation step (see Sec. S1 D 2).

Using the "initial step" process means that once a critical event occurs, the previous interval is forgotten and segmentation restarts as in Sec. S1 D 1. To decide whether an event can be considered critical, we require that i) the similarity corresponding to the last Δt is greater than 0.95 of the peak similarity and ii) we have not reached the end of the dataset.

An example of critical events can be seen in main text Fig. 1(a-c). Each interval boundary occurs at a critical event.

S2. DATASET DESCRIPTIONS

A. Periodic toy model

This model creates data with evolving timescales while having a uniform event rate. The latter means that at every point in time, there is the same expected value of the number of events. Our method does not group the data in intervals using the overall event rate, it detects intervals based on the *identity* of events at a given time. In this model, the characteristic active events change slowly over time, but at a varying rate. This adds the "evolutionary

Number of events	43599
Number of distinct events	1275
First event time	1999-05-11
Last event time	2002-06-21
Total count/weight of events	43599

TABLE S1: Basic properties of Enron dataset (core network)

Number of events	802481
Number of distinct events	207071
First event time	1998-05-26
Last event time	2002-07-11
Total count/weight of events	2933183

TABLE S2: Basic properties of Enron dataset (full data)

timescales" that our method detects. When the timescales evolve faster, intervals are shorter, and when timescales evolve slower, intervals are longer.

In this periodic toy model, a universe of $N = 1000$ event IDs are created. Upon initialization, each event is independently placed into an activated status with probability q. At each timestep, each active ID emits an event with probability p. This p produces the short-term repetition, on a timescale of $1/p$. Also, at each timestep (and before event creation), a fraction $c(t)$ of event IDs are picked. Each of these IDs has its active status updated, being made active with probability q regardless of its previous state. This provides the long-term change: on average, each active event tends to lasting on the order of $1/c(t)$ time steps until it is deactivated. This preserves the mean total number of activated IDs as qN at all times. At each timestep, on average pqN events are emitted.

The changeover rate $c(t)$ is periodic, however, obeying

$$c(t) = c_0 + c\left(\frac{1}{2} - \frac{1}{2}\cos\left(\frac{2\pi t}{\tau}\right)\right), \tag{S1}$$

with c_0 being the minimum changeover rate and, c being the scale of changeover, and τ being the period. Thus, at some points, the IDs of the expressed events is changing more rapidly than at other times, and this is the cause of longer and shorter intervals.

When there is a critical event at time t_{crit}, we exceptionally set $c(t_{\text{crit}}) = 1$ at that time. This produces a state of the system decorrelated from the previous time. According to the model, this changeover occurs before the timestep, which matches with placing a new interval at t_{crit} in our half-open segment convention. However, the total universe of event IDs stays the same, and there is no statically observable change in behavior.

In this work, we use $N = 1000$, $\tau = 500$, $p = 0.2$, $q = 0.2$, $c_0 = 0$, and $c = 0.01$.

B. Enron

The Enron Email Dataset consists of emails of approximately 150 senior managers of the Enron Corporation, which collapsed in 2001 after market manipulation was uncovered, leading to an accounting scandal [2]. This dataset was made public during the legal investigation concerning the Enron Corporation, which ended up with the bankruptcy and collapse of this corporation. Here, each event is an email communication sent or received by any of the senior managers who were subsequently investigated. During the time recorded in the dataset, there were major structural changes in the company, executives were hired and fired and new products were launched. This exogenous information was sometimes also reflected in the data as changes in volume and in the identities of active events.

We have all bidirectional email communication to and from each key person at a resolution of one day. An event is the unordered pair (source, destination) of each email. Our particular input data is already aggregated by day, therefore each event is only present once each day, with a weight of the number of mails that day. Table S1-S2 list the basic properties and Table S3 lists the major events of this dataset. In Fig. S1, we show the full Enron data. This includes one event for every message sent, even to other executives.

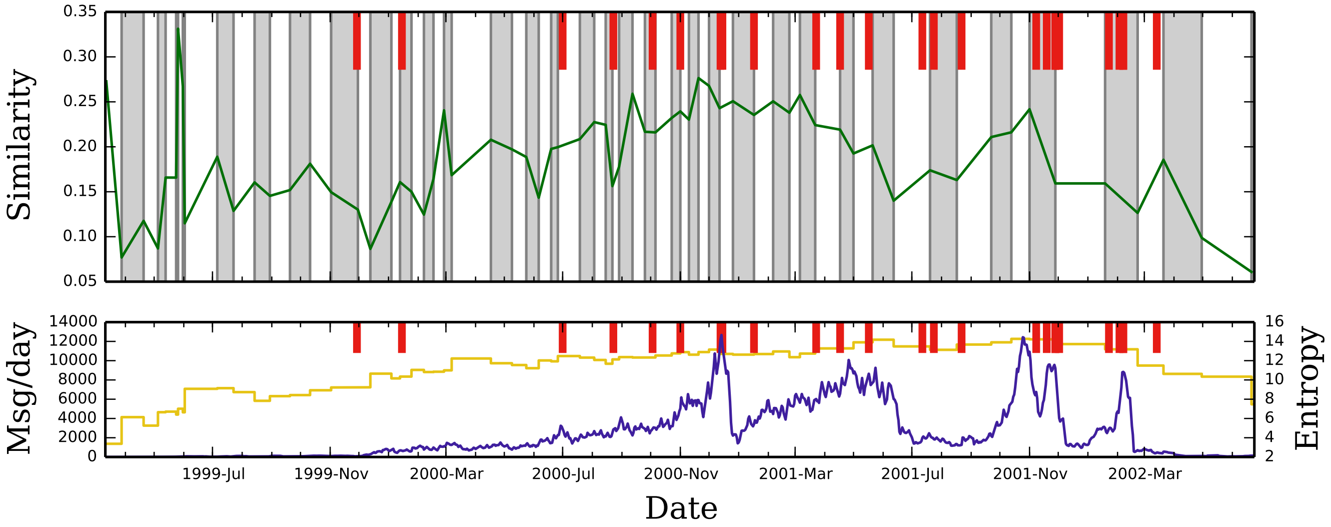

FIG. S1: Slicing of the full Enron dataset. The format of this figure is as in Fig. 2 from the main text. Thick red bars at the top of the plots correspond to actual dates of major events. This is the slicing of the whole dataset, not just the links between executives.

Date	Event
1999-11-29	Launch of enron online
2000-01-15	Launch of EBS
2000-07-01	EBS-blockbusters partnership
2000-08-23	Stock all-time high
2000-10-03	Enron attorney discusses Belden's strategies
2000-11-01	FERC exonerates Enron
2000-12-15	EBS \$53m 'profit'
2000-12-13	Skilling announced to be CEO
2001-01-17	Blackouts in CA
2001-03-23	Enron schedules conference call to boost stock
2001-04-17	The 'asshole' call
2001-05-17	Schwarzenegger, Lay, Milken meeting
2001-07-12	Quarterly conference call
2001-07-24	Skilling meets with analysists and investors in NY
2001-08-22	Watkins raises accounting irregularities
2001-11-08	Dynegy agrees to buy enron
2001-11-19	Enron restates its third quarter earning
2001-11-28	Enron shares plunge below \$1
2001-11-28	Dynergy deal collapses
2001-12-02	Enron files for bankrupcy
2002-01-23	Stephen Cooper takes over as Enron CEO
2002-02-03	Lay cancels Senate committee appearance
2002-02-07	Fastow, Kopper, Lay invoke the Fifth
2002-02-07	Skilling and Watkins testify
2002-03-14	Arthur Anderen LLP indicted

TABLE S3: Major events of the Enron collapse

1. *Comparison with the cpdetect method*

The *cpdetect* method is run as described in Ref. [4]. A part of this process is to pre-aggregate all data into windows of one week size, and thus any changepoints are *a priori* limited in their accuracy. This process is not necessary in our work, and one possible use of our method is providing initial intervals for other methods such as cpdetect.

The output from our method and cpdetect is not directly comparable. The cpdetect method produces only significant changepoints, while our method should produce snapshots distributed throughout the entire time period.

Number of events	1881152
Number of distinct events	51466
First event time	2004-08-01 00:26:38
Last event time	2005-07-14 17:41:19
Total count/weight of events	1881152

TABLE S4: Basic properties of the Reality Mining dataset

Date	Event
2004-09-08	First day of classes
2004-10-18	Sponsor week end
2004-10-25	Sponsor week start
2004-11-08	Exam week start
2004-11-12	Exam week end
2004-11-25	First day of thanksgiving
2004-12-09	Last day of classes
2004-12-13	Exams start (4 days)
2005-01-03	Independent activities period start
2005-02-01	Start of spring classes
2005-03-19	Start of spring break
2005-03-28	End spring break
2005-05-12	Last day of classes
2005-05-16	Exams start
2005-06-03	Commencement

TABLE S5: Major events of the Enron dataset

However, we would expect that the similarity J is lower than average at each changepoint. If so, it will indicate that our method can show at least the major underlying dynamics of the system. We show this with a hypothesis test. To do this, calculate a test statistic which is our mean (interpolated) similarity value corresponding to each change point found by cpdetect. We randomize the times of change points by picking times uniformly from our entire time range of changepoints, which shuffles the change point sequence. We do this 1×10^4 times to compute a distribution of our test statistic in the random case. We find that our actual value of X is significant at $p = 0.0033$. This means that our choice of similarity does correlate with the evolutionary timescale of the system. If one looks at low values of J, one can find changepoints, and our process of maximizing J finds self-similar intervals.

C. Reality mining dataset

The Reality Mining dataset covers a group of persons affiliated with the MIT Media Lab who were given phones which tracked other devices in close proximity via the Bluetooth personal area network protocol [1]. We say an event is every ordered pair of (personal_device, other_device). We only have data from the personal devices of the 91 subjects who completed the experiment, not full ego networks. The data contains 1881152 unique readings from 2004 August 8 until 2005 July 14, being most of them centered in the middle of the academic year. Table S4 lists basic properties and Table S5 lists the major events of this dataset. The major events are from the MIT academic calendar or internal Media Lab events, with which most subjects were affiliated [3].

As expected, we find many more segment intervals than major events. In the slicing, we can see that in the first weeks, where students are still relocating and meeting new people, the change in the events' identities is fast, thus the intervals are shorter. On the other hand, the interactions throughout the rest of the academic year are pretty stable, and the intervals are sized accordingly. Additionally, we see that several key events are detected. The major Thanksgiving holiday (Nov 25) is detected a day early, as people begin traveling. There are intervals before and after the start of the December exam week, and then a stable period after that when people are on holiday until the university opens again (Jan 3). After that, we see segment boundaries that exactly align with the beginning of classes (Feb 1), the beginning of spring break (Mar 19), and the last day of classes (May 12). All of this can be detected despite the fact that many subjects are research staff, who are not bound by any academic calendar.

	Entire data	Game 1	Game 2
Number of events	401849	87900	120123
Number of distinct events	8650	2660	3161
First event time	2015-04-30 16:08:42	2015-05-05 19:45:00	2015-05-06 19:45:00
Last event time	2015-05-08 01:59:45	2015-05-05 23:44:59	2015-05-06 23:44:59
Total count/weight of events	401849	87900	120123

TABLE S6: Basic properties of the UCL dataset. All times are in CEST, the local timezone of the matches.

Date	Event
2015-05-05 20:45	Game 1 begins
2015-05-05 20:50	Yellow card for Bonucci (Juventus)
2015-05-05 20:53	Goal by Morata (Juventus)
2015-05-05 21:12	Goal by Ronaldo (Real Madrid)
2015-05-05 21:29	First half ends
2015-05-05 21:45	Second half begins
2015-05-05 21:58	Goal by Tévez (Juventus)
2015-05-05 22:33	Game 1 ends
2015-05-06 20:45	Game 2 begins
2015-05-06 21:29	First half ends
2015-05-06 21:45	Second half begins
2015-05-06 22:17	Messi (Barcelona) scores
2015-05-06 22:20	Messi (Barcelona) scores
2015-05-06 22:34	Neymar (Barcelona) scores
2015-05-06 22:34	Game 2 ends

TABLE S7: Major events of the first leg of the Champion's League semifinals

D. UCL hashtags

In this dataset, we scrape Twitter for all hashtags containing `#UCL`, referring to the UEFA (European) Champions League tournament. We scrape between the dates of 30 April 2015 and 08 May 2015, covering the first leg of the two semifinal games of the tournament. An event is any hashtag co-occurring with `#UCL`, except `#UCL` itself. If there are multiple hashtags in the same tweet, each counts as one event. Thus, events (hashtags) reflect what people are talking about in conjunction with the tournament. All hashtags are interpreted as UTF-8 Unicode and case-normalized (converted to lowercase) before turning into events. Table S6 lists basic properties and Table S7 lists the major events of this dataset.

In the slicing (main text Fig. 5(a)), we see much intervals segments during the games of May 5 and May 6 than in the rest of the dataset. This corresponds to the must faster turnover of interesting topics during the game. In addition, we can spot the games that all four teams played in their respective national championships on the previous weekend of May 2-3, which trigger as well some discussion on the forthcoming Champions League matches. If we focus our attention on the first of the two games (main text Fig. 5(b)), we observe a shorter segment size at the beginning of the game and during the three goals at times 20:53 (Juventus), 21:12 (Real Madrid), and 21:58 (Juventus), compared to the rest of the match. In particular, we notice a more noticeable effect when the second half started with the tied game at 21:45, and when the third goal is scored, because it was a penalty kick which took Juventus to the lead. Game 2 was a lopsided 3-0 victory for Barcelona against Bayern Munich. Given the one-sided game, the analysis shows a fairly uniform pattern throughout the game, until the goals are scored at 22:17, 22:20, and 22:34. The game ends right after the last goal. In the initial part of the game, the dynamics continually slowed down as time goes on without major events. Immediately after the game, the audience reacts much more strongly than in the last game, probably because at that point one starts speculating about the possible finalists of the competition.

The detail of the second game is presented in Fig. S2. There is a flurry of activity at the start, but as the game progresses without goals dynamics slow down. At the end of the game, there are three goals (beginning at 22:17). At this point, the system becomes extremely active with a very diverse and rapidly changing topics, and intervals are very short for a while.

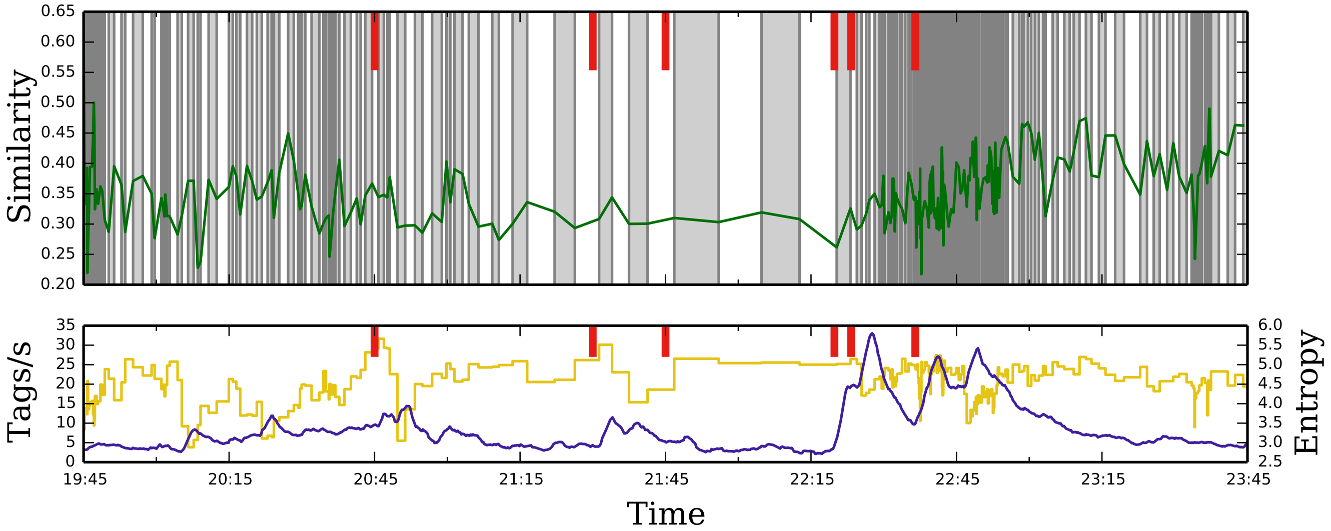

FIG. S2: Second semifinal of the UEFA Champions League 2014-2015, between Barcelona and Bayern Munich. Game times are the same as in main text Fig. 5(b) in the main text. This game was one-sided with goals made by only one team. Consequently, internal structure for the slicing is relatively uniform and slowing down with time, especially since all goals were scored within the last 20 minutes of the game. Nevertheless, we can detect interval boundaries for halftime, and after the three goals are scored (22:17, 22:20, and 22:34), we have a very high turnover rate of discussion topics.

---

[1] Nathan Eagle and Alex Pentland. Reality mining: sensing complex social systems. *Personal and ubiquitous computing*, 10(4):255–268, 2006.

[2] Bryan Klimt and Yiming Yang. The enron corpus: A new dataset for email classification research. pages 217–226. Machine learning: ECML 2004, 2004.

[3] MIT. MIT academic calendar 2004-2005. The Internet Archive Wayback Machine, `https://web.archive.org/web/20041009182037/http://web.mit.edu/registrar/www/calendar.html`, 2004.

[4] Leto Peel and Aaron Clauset. Detecting change points in the large-scale structure of evolving networks. AAAI Conference on Artificial Intelligence, 2015.
